# Can the Biophilia Hypothesis Be Applied to Long-Duration Human Space Flight? A Mini-Review

**DOI:** 10.3389/fpsyg.2021.703766

**Published:** 2021-09-09

**Authors:** Brittany N. Neilson, Curtis M. Craig, George C. Altman, Alexandra T. Travis, Joseph A. Vance, Martina I. Klein

**Affiliations:** ^1^Stress, Workload, and Performance Laboratory, Department of Psychological Sciences, Texas Tech University, Lubbock, TX, United States; ^2^HumanFIRST Laboratory, Department of Mechanical Engineering, University of Minnesota, Minneapolis, MN, United States

**Keywords:** biophilia hypothesis, restorative effect of nature, restorative environments, long-duration spaceflight, aerospace systems

## Abstract

The International Space Station (ISS) has around 3–5 crew members on-board at all times, and they normally stay on the ISS for about 5–7months in duration. Since March 2020, 170 long-duration space missions have occurred on the ISS. Thus, long-duration space missions are an integral part of space exploration and will only continue to expand in duration as missions to the Moon and Mars are on the horizon. However, long-duration space missions present several challenges to human crew members. Most of these challenges have been associated with physiological adaptation to microgravity, including motion sickness, muscle atrophy, and cardiovascular deconditioning. While not as well-studied, another major factor to consider when planning long-duration space missions is the psychological impact of the environment on the astronauts. Astronauts living in space will be unable to access natural landscapes and other environments found to have restorative effects on psychological stress and overall well-being. On top of being unable to access these restorative natural environments, astronauts will also be exposed to the stressful, unfamiliar environment of space. The purpose of this mini-review is to first summarize the literature related to stressors associated with space. Next, an overview of the large breadth of literature on the biophilia hypothesis and restorative environments will be provided, as these may serve as relatively simple and cost-effective solutions to mitigate the stress faced during long-duration space missions. Lastly, considerations related to the design of such environments in a space capsule as well as future directions will be presented.

## Introduction

The ancestors of humans lived in nature for millions of years, using its resources for food, water, shelter, and protection. In many modern-day societies, urbanization and technological advancements have reduced nature’s role in survival. Instead of being a nomad foraging through natural environments to find our basic needs, our species now mostly congregate together in urbanized environments that utilize mass production to provide us with resources needed for survival.

While these industrial and technological advancements have many benefits, our modern life has created a deficit of nature, and it is presently unknown the long-term impact that this may have on us. A theory called *biophilia* (Wilson, 1984) suggests that humans have an innate propensity for nature, and there may be an evolutionary benefit to such a propensity. A large body of empirical research has supported the notion that being in natural environments compared to urbanized environments has a breadth of psychological benefits (see Berto, 2014, for review). Thus, a disconnection from nature may have a real and profound impact on our overall well-being.

From this, there has been recent interest in making urban living “green” by adding greenspaces (e.g., vegetation, trees, gardens, plants, man-made lakes, and parks) to surrounding offices, neighborhoods, and buildings as well as inside urban spaces (Kabisch et al., 2015; Hunter et al., 2019). Even the term “green office” is now common; office spaces that include plants, sunlight, window views, and the ability to open windows for fresh air are thought to reduce stress, improve mood, and ultimately, enhance work productivity and job satisfaction (Lottrup et al., 2013, 2015). Even during restrictions due to the COVID-19 pandemic, recommendations for maintaining access to greenspaces were presented by healthcare professionals (Slater et al., 2020), as they presumably recognized the positive association between spending time in these spaces and one’s health. While current efforts to make urban living “greener” are important, especially as we continue to urbanize, there are other areas of technological advancements that could benefit from incorporating a biophilic design approach.

One such area is the human-nature disconnection that is inherent in long-duration space missions. As human space travel moves further from the Earth, the amount of time spent living in a built environment (i.e., space shuttle or capsule) will increase. Thus, the detriments to human well-being that have been documented in more urbanized living will likely be worsened in space, as there are additional stressors associated with the extreme environment of space. The purpose of this mini-review is 2-fold: (1) to highlight additional and potentially overlooked stressors associated with long-duration space missions that biophilia would predict and (2) to propose methods based upon related empirical research for incorporating green design to space environments. The mini-review was conducted using OneSearch offered through the Texas Tech University’s library, Google Scholar, and the NASA.gov website. Priority was given to recently published research as well as research on greenspaces that can be implemented practically in outer space. The overarching aim is that this mini-review sparks new developments in space research, inspired by the field of biophilia that will ultimately enhance the well-being and safety of those brave individuals who will embark on future journeys to the Moon, Mars, and beyond the Earth.

## Humans in Space and Associated Stressors

Reason (1974) introduced his book, “Man in Motion: The Psychology of Travel,” with the following passage: “It is sometimes said by reluctant air travelers that if man had been intended to fly, he would have been provided with wings of his own. But this is only part of the truth. If man had been intended to move about by any means than under his own power, he would have been built to an altogether different set of specifications” (p. 1). As he articulated, any form of human travel beyond foot is diverging from human evolution. These advancements in modes of transportation have humans passively moving at much faster speeds compared to walking. While increased speed of travel is more efficient, it has negative consequences (e.g., motion sickness and spatial disorientation; Reason, 1974).

Human space travel is arguably the greatest advancement among travel modes to this day. However, there are many additional and unique side effects associated with space travel, including physiological adaptation to microgravity, which in turn disrupts the vestibular system, creates muscle atrophy, and impairs cardiovascular functioning, namely cardiac deconditioning that results in reduced autonomic nervous system responses (Aubert et al., 2005). There are also neurological and cognitive impacts attributed to microgravity and other space-relevant factors (for reviews, see De la Torre, 2014 and Clément et al., 2020); decrements in cognitive performance are not always present (Kelly et al., 2005) but have included disruptions in perceptual-motor skills, reduced tracking performance, and interference with dual-tasking or performing two tasks at once (Kanas and Manzey, 2008).

While the impact of microgravity has been well-researched, there are many other environmental stressors (e.g., exposure to extreme temperatures, in-flight noise, radiation, and circadian rhythm disruption; Thirsk et al., 2009) and psychological stressors (e.g., team dynamics, mental fatigue, isolation, and confinement with regard to newer, smaller space capsules; Kanas and Manzey, 2008; Musso et al., 2018) associated with space travel. The combination of all of these variables associated with space travel can create a significant amount of overall stress, or what has been noted in the space psychology literature as “non-specific stress” (Kanas and Manzey, 2008).

Monitoring and understanding an operator’s stress state are challenging because observing operator performance does not necessarily specify their stress state. High stress states can harm performance (Henderson et al., 2012), but skilled individuals have compensatory strategies and behavior for mitigating stress and maintaining superior performance (Hockey, 2011). While performance may or may not be overtly impacted during routine operations, a stressed state leaves the operator vulnerable to a disastrous mistake during unexpected situations. For example, the task of driving can be stressful in heavy traffic. A compensatory behavior for a skilled driver may be to reduce distractions (e.g., lower stereo volume) and allocate all of one’s attention to the driving task. Driving performance may not be impaired, but the high stress state is present and may cause mistakes during unusual events (e.g., avoiding another vehicle driving the wrong way). This is important to consider in spaceflight, as astronauts are typically highly skilled performers, which may disguise their stress states.

The need to reduce stress is especially critical for long-duration spaceflight missions, where there will be major consequences for stress-induced errors and few opportunities for recovery. Influencing or modifying the astronaut’s environment may be one approach to mitigate stress and enhance performance. Research related to the biophilia hypothesis has supported the important role that natural environments can play in reducing stress and improving attentional capacity. A review of the biophilia hypothesis and empirical research supporting nature’s impact on stress states and cognitive performance is presented. Finally, some ideas on implementation in spaceflight are presented.

## The Biophilia Hypothesis

The biophilia hypothesis is that most people want to attend, be around, and have a positive response to nature, and that there is a genetic basis for this behavior (Wilson, 1984). For this to be true, a preference for nature would have to engender some survival or reproductive advantage to the species at some point in evolutionary history. In a review of evidence for biophilia, Ulrich (1993) observed that open spaces, water proximity, green vegetation, and flowers allowed for the ability to evade predators and have better access to water and food. Therefore, these spaces should be and are more preferred by people. If there is a general adaptive positive effect of nature environments, as implied by the biophilia hypothesis, the consequence of being in nature would be 3-fold: (1) Higher liking or preference and approach behavior, (2) better stress recovery and/or recuperation from cognitive depletion, and (3) enhanced higher-order cognition (Ulrich, 1993).

Kellert (2008) has attempted to translate this hypothesized preference for nature to design elements for the built environment. These include, but are not limited to, environmental features (e.g., Earth-like hues, water, and plants), natural shapes and forms (e.g., seashells and tree shapes), natural patterns and processes (e.g., fractals, portrayal of aging, and sensory variability), light and space (e.g., natural light and light pools), and place-based relationships (e.g., features unique to a location, such as indigenous materials and historical connection). Each category comprised numerous subcategories to better delineate features of nature and how they can be implemented. In a review of the evidence of benefits for these biophilic features within built environments, Gillis and Gatersleben (2015) observed benefits of environmental features (e.g., water and plants) but limited evidence for the benefits of other biophilic features like natural materials and processes (e.g., wood). The measures used as evidence were primarily indicators of preference or metrics related to recovery of stress and cognitive decline.

Interestingly, across a breadth of research involving participants varying in age, location, and culture, there continues to be evidence suggesting that nature environments have a positive impact on stress and cognition. The relationship between natural environments, reduced stress, and improved cognition is referred to in this paper as the “restorative effect” and has been empirically demonstrated (Ulrich et al., 1991; Hartig et al., 2003; Berman et al., 2008 , 2012; Bowler et al., 2010; McMahan and Estes, 2015; Ohly et al., 2016; Kotera et al., 2020). Typically, these studies compare the impact of environments deemed to be restorative (usually nature) to environments considered non-restorative (usually urban settings) on different outcome variables. The restorative environments are typically presented as one of the following approaches: (1) immersion in the real environment (Hartig et al., 2003; Berman et al., 2008), (2) exposure to videos or digital images (Ulrich et al., 1991; Berman et al., 2008), or (3) views from windows (Ulrich, 1984). The outcome variables on which the restorative effect has been tested are extensive, including attention and other cognitive measures (Ohly et al., 2016), self-reported mood and stress assessments (Bowler et al., 2010), and physiological stress correlates (Ulrich et al., 1991).

It should be noted that there are several proposed mechanisms to explain these aforementioned benefits (see Ulrich et al., 1991 and Kaplan, 1995 for prominent mechanisms and Berto, 2014 for review), but there is a lack of clarity regarding the contexts under which they apply and the impact that individual, cultural, and societal differences may play. In general, the biophilia hypothesis explains the human connectedness to nature on a macro-level, but the process by which experiencing nature mitigates stress and improves cognitive functioning is not entirely agreed upon and understood. Future research should aim to better develop a model to explain the intricacies in the relationship between nature exposure and the various outcomes related to well-being, as this will inform which contexts a restorative intervention could be applied (e.g., which situations in the space environment would be appropriate to implement a break involving nature) and to whom (e.g., some individuals may be more responsive to the restorative effect than others).

Even more so, the interrelationship between biophilia and spaceflight has been understudied. While Kanas and Manzey (2008) briefly allude to potential issues associated with the “Earth-out-of-view phenomenon” during prospective Mars missions, the primary area in which a similar relationship has been considered has been with the “overview effect,” which is the shift in perspective and experience of awe that occurs when astronauts see the Earth from space. Astronauts who experience this “overview effect” report higher positive attitudes toward nature, a greater concern for environmental issues, and increased positive personal behaviors like adopting more sustainable diets, shopping habits, water usage, and so on (Voski, 2020). However, this effect is only tangentially related to biophilia, is only available for local spaceflight around the Earth, and is not necessarily useful for the later phases of long-duration flights.

Without clear research connecting biophilia and spaceflight, one must make limited recommendations based on effective biophilic designs here on Earth. Given that Gillis and Gatersleben (2015) observe reliable effects of environmental features on stress recovery, this suggests that designers and engineers that are interested in implementing biophilia in space should focus on types of environmental features outlined by Kellert (2008). These types include colors consistent with the natural world (e.g., Earth tones), water, natural ventilation, sunlight (and replication of sunlight), plants, animals, views and vistas, and so on, although the exact manner of implementation of these features in spaceflight will require further work.

## Discussion

It stands to reason that some of the green spaces or natural interventions that are beneficial on Earth may not be feasible in outer space. For example, access to a large natural space, such as a park or forest, is obviously impossible during space travel. Still, there may be some methods of translating natural elements to space environments. These methods will need to meet the constraints of a spacecraft (e.g., limits on physical space, power generation, and energy storage) and will certainly not represent a solution to all human performance and stress-related issues. Further, more research is needed to better understand individual differences that may impact the effect of such methods.

### Methods of Implementation in Space

#### Virtual Reality

One potential way of replacing a break in large natural settings comes in the form of virtual environments, achieved through the use of virtual reality (VR) headsets. In fact, NASA’s current research on possible implementations of virtual reality (Hille, 2020) at least supports the notion that VR is feasible on a logistical level. VR headsets and the necessary hardware to use them are also relatively manageable in terms of size, making it a reasonable method of implementing nature into space missions of any length, though their power consumption may be an issue that requires investigation. Preliminary research on the usage of VR has shown that environments with natural elements can reduce stress and anxiety (Yin et al., 2020). Virtual natural spaces have also been shown to be perceived as restorative as natural outdoor environments and even potentially evoke similar engagement, interest, and positive affect akin to natural outdoor environments, as indicated by physiological correlates and self-reports (Browning et al., 2020).

#### Artificial Windows

While a window view of an actual natural environment is impossible in space, artificial windows may be a reasonable alternative. A study involving a prototype system that created a realistic 3-D natural window view had a positive impact on participant’s mood (Radikovic et al., 2005), albeit more traditional 2-D artificial windows have had less success (Kim et al., 2018). While artificial windows may be a promising alternative to a real window view, given that they are relatively cheap and have been easy to implement on Earth, more research is required into both the efficacy and practicality for usage on a spacecraft.

#### Vegetation

Potted plants or plants within gardens could be a feasible biophilic addition to a spacecraft, depending on the amount of usable space. NASA is already investigating the possibility of plants in space through programs such as the Vegetable Production System (Massa et al., 2016). In fact, others have advocated for engaging in gardening during long-duration spaceflight as a method to mitigate stress, promote prosocial behavior, and increase brain activation in various regions (Odeh and Guy, 2017). The presence of indoor plants has also been shown to have a positive impact on attentional capacity, which is important to sustain during astronaut’s day-to-day duties, like monitoring the spacecraft, performing experiments, and maintaining equipment (Raanaas et al., 2010).

#### Digital Media

Digital nature images are among the cheapest and most efficient methods of introducing greenery to passengers aboard a spacecraft; however, they are generally less effective than more immersive methods. Interestingly, incarcerated men who were exposed to digital nature images and sounds for just 12min, twice a day, lasting for 10 consecutive days reported significantly less stress and had a reduction in physiological stress (Nadkarni et al., 2021). Since the amount of isolation and stress among astronauts may be similar to the participants in the aforementioned study, the results are promising with regard to digital media involving nature serving as a potential way to reduce stress. More broadly, cognitive performance improvements have been demonstrated after viewing natural images compared to urban images (Berman et al., 2008). Additional studies have also found that certain “awe-inspiring” natural images, such as waterfalls, or natural images containing lakes, oceans, and rivers could have an even greater impact on mood and prosocial attitudes than standard nature images, suggesting the specific type of natural image could be of importance (White et al., 2010; Joye and Bolderdijk, 2015). In a similar vein, natural sounds have been used in many studies to evoke a restorative effect. One study found improved mood and task performance when presented with nature sounds (Van Hedger et al., 2019). Perhaps, digital images and audio can be used simultaneously to create a stronger restorative effect for astronauts.

## Conclusion

The purpose of this mini-review is to present a novel challenge associated with long-duration space missions as well as potential countermeasures that could aid in resolving such a challenge. The challenge is 2-fold: Astronauts will be expected to inhabit an extreme environment full of stressors for a long duration, and they will be removed from their natural environment on Earth, which may have negative side effects from a lack of access to biophilic/restorative environments. Evidence was presented to support such concerns for these challenges. Several potential methods for implementing a “green”/biophilic/restorative design to a space shuttle or capsule were presented. These are presented, not to serve as a cure-all, but rather to highlight areas of research that need greater attention.

## Author Contributions

BN was the lead author who formulated the idea and made sure the document was cohesive. BN also wrotethe introduction and conclusion. BN and JV wrote the stressors in space section. CC, AT, and MK wrote the biophilia section. GA wrote the implementation section. All authors contributed to the article and approved the submitted version.

## Conflict of Interest

The authors declare that the research was conducted in the absence of any commercial or financial relationships that could be construed as a potential conflict of interest.

## Publisher’s Note

All claims expressed in this article are solely those of the authors and do not necessarily represent those of their affiliated organizations, or those of the publisher, the editors and the reviewers. Any product that may be evaluated in this article, or claim that may be made by its manufacturer, is not guaranteed or endorsed by the publisher.
